# Stigma, Health and Well-Being

**DOI:** 10.3390/ijerph17207615

**Published:** 2020-10-19

**Authors:** Chung-Ying Lin, Hector W. H. Tsang

**Affiliations:** 1 Institute of Allied Health Sciences, National Cheng Kung University Hospital, College of Medicine, National Cheng Kung University, Tainan 701, Taiwan; 2 Department of Rehabilitation Sciences, The Hong Kong Polytechnic University, Hung Hom, Hong Kong, China; Hector.Tsang@polyu.edu.hk

**Keywords:** discrimination, prejudice, quality of life, stigma, well-being

## Abstract

In order to understand the phenomenon of stigma in different populations (e.g., different ethnicities, different diseases, and different conditions), this Special Issue collects papers from around the world to illustrate the stigma phenomenon. After a rigorous process of peer review, a total of 24 papers were published and included in the Special Issue. These papers were contributed from different continents and countries, including the Americas (e.g., United States), Europe (e.g., Finland), and Asia (e.g., Saudi Arabia). Therefore, the diversity of ethnicity was ensured in the Special Issue. Moreover, these papers address different stigmatized populations/conditions (e.g., mental illness, obesity, public housing, homosexuality, and transgender). The most discussed populations were those with mental illness and those with obesity/overweight. However, additional evidence on the stigma topic is still needed. Specifically, future studies could consider the following directions to explore in depth the issues of stigma in different populations: (1) using longitudinal designs to understand the temporal or causal relationship between stigma and other related psychosocial factors; (2) designing treatment programs to fight stigma—this could be carried out in healthcare providers, healthcare trainees, the public, caregivers, and the stigmatized populations.

## 1. Introduction

Although the modern world has enhanced the quality of healthcare and increased the successful rate of treatment due to the health technologies and available medications available, some psychosocial issues related to diseases remain unresolved. Indeed, the literature shows that populations with different disabilities have increased life expectancy [1,2]; however, their psychosocial health seems not to be well taken care of in our communities.

Among these psychosocial health issues, stigma seems to be one of the most important issues that should be tackled to avoid its tremendous and negative impacts on health promotion [3]. According to the literature, stigma can be conceptualized as the co-occurrence of the following: (i) labeling and distinguishing differences; (ii) stereotyping the individuals who are stigmatized as different in a negative way; (iii) separating the individual according to the labeling (i.e., differentiating people who are labeled from those who are not (e.g., using the term of “us” for unlabeled individuals and “them” for labeled individuals)); (iv) having negative emotional reactions (e.g., anger and fear) toward the individuals who are stigmatized or labeled; (v) discriminating the group which is labeled and letting the labeled group experience status loss, and (vi) unfolding the aforementioned processes via economic, social, and political ways to decrease the power of the labeled population [4,5]. Accordingly, a variety of forms and types of stigma have been proposed: public stigma, experienced stigma, perceived stigma, and self-stigma (i.e., internalized stigma) [6]. Ample literature also shows that any type of stigma poses a great threat to well-being and health through different routes (e.g., lost opportunities in treatment availability, employment, education, and living) for those who are stigmatized [3,6,7,8]. Therefore, the individuals who are stigmatized are at high risk of having low quality of life. In order to resolve the complex issues of stigma, the link between stigma and health should be substantially investigated. More evidence is thus needed to assist healthcare providers who work in the field in understanding the stigma issue among populations worldwide.

## 2. The Aims and Contributions of the Special Issue

In this Special Issue, we intend to address the knowledge gap in the understanding of stigma and how it relates to different aspects of health outcomes across different populations. Therefore, we have invited international submissions of papers and report the state-of-art information on stigma issues. We are glad that a total of 24 original papers have been peer reviewed and published in the Special Issue. The aims of the Special Issue are fully addressed by the contributed papers. Specifically, the papers include diverse populations in ethnicity (e.g., Caucasians, Vietnams, Taiwanese, Korean, Hong Kong people, and Saudi Arabian), disability (e.g., physical disability and mental health problems), conditions (e.g., transgender, weight problems, and caregiving), and human developmental stages (e.g., adolescence and young adults). Detailed information of the contributions can be found in the following section (i.e., 3. Findings of the Contributions in the Special Issue). Therefore, we are confident that the collection of the Special Issue achieves our original intention of filling the literature gap on stigma.

## 3. Findings of the Contributions in the Special Issue

### 3.1. Stigma on Obesity

The findings of the contributions in the Special Issue are summarized in Table 1. Lipowska et al. [9] compared the perceived stigma between university students from Poland and those from Vietnam to illustrate the cultural differences in body stigma. Their findings indicate that Confucian concepts of modesty and shame may impact university students’ concern about their bodies; therefore, university students from Vietnam were more vulnerable than those from Poland to criticisms of their bodies. Similar to the body shape issue, Lin et al. [10] assessed two types of weight stigma (perceived weight stigma and weight-related self-stigma) in 464 junior high school students in Taiwan. Their findings indicate that adolescents with overweight using a self-reported body mass index had higher levels of perceived weight stigma and self-stigma but not public stigma; similar findings were revealed in adolescents with self-perceived overweight versus those with self-perceived non-overweight. In addition, Lin et al. [10] found that higher levels of perceived weight stigma were associated with higher levels of anxiety among adolescents with overweight regardless of whether this was actual or self-perceived.

Fung et al. [11] incorporated the weight-related self-stigma concepts into the well-known Theory of Planned Behavior model among Hong Kong university students. With the satisfactory model fit in the structural equation modeling, they found that higher levels of weight-related self-stigma were associated with lower levels of physical activity among university students who were overweight (n = 104). However, such negative association was not observed among those who were not overweight (n = 221). In addition to the finding regarding the association between weight-related self-stigma and healthy behaviors, an association between experienced weight stigma and unhealthy behaviors has been found. Wu et al. [12] recruited 166 Asian Americans, with most of them being the first generation to understand the association between experienced weight stigma and binge eating behaviors. The participants were defined as overweight according to the body mass index cutoff for Asians (i.e., body mass index > 23 kg/m^2^). After controlling the effects of racism and general stress, Wu et al. [12] concluded that experienced weight stigma is an independent factor associated with binge eating behaviors for those who may have a weight problem.

Panza et al. [13] additionally studied weight stigma in a special population of sexual minority women with overweight/obesity (i.e., body mass index over 25 kg/m^2^) in the United States. Although their sample size was relatively small, high rates of experienced weight were observed among this sample. In addition, their identity of sexual minority was co-occurrent with their perceived and experienced weight stigma. Therefore, studies with a large size are needed to deeply understand this situation for sexual minority women. 

### 3.2. Stigma on Mental Illness

In addition to obesity, mental illness (including anxiety, attention-deficit/hyperactivity disorder (ADHD), autism spectrum (ASD), dementia, and depression) is another disease/condition that is largely studied with stigma. Luís et al. [14] investigated 214 male adults having a heterosexual relationship with a reproductive-age partner who were suffering from postpartum mood and anxiety disorder in Coimbra. They applied structural equation modeling to quantify the association between stigma (possibly self-stigma) and intentions to recommend professional help-seeking. Their results indicate that the male adults with higher levels of (self-)stigma had lower intentions to recommend professional help-seeking to their partners. 

Rodríguez-Almagro et al. [15] used a mixed-methods study to understand nursing students’ attitudes toward mental illness in Spain. Through data from 359 (quantitative study) and 30 (qualitative study) nursing students, associations between lowered public stigma and elevated positive attitude toward people with mental illness were found. Additionally, nursing students who were in a higher year of study (especially third year or above) had lower levels of public stigma than those who were in a lower year of study. Riffel and Chen [16] also studied the students’ attitudes toward mental illness. However, Riffel and Chen [16] investigated more than nursing students and they only used a qualitative design to understand students’ attitudes. Through semi-structured interview and a qualitative description method, 18 students from nine health programs in a Canadian University revealed their attitudes toward mental illness. The findings indicate that most students had good knowledge of mental health and they showed positive behaviors when interacting with people with mental illness. However, stigmatizing attitudes and unpreparedness to work with people with mental illness were observed in some students.

Chang et al. [17] recruited 400 caregivers who were taking care of a child diagnosed with attention-deficit/hyperactivity disorder (ADHD) in Taiwan. Their findings indicated that caregivers’ affiliate stigma is a significant factor associated with their unfarmable attitudes toward the ADHD diagnosis and related treatments. In addition, the aforementioned association was not moderated or confounded by sociodemographic factors and ADHD symptoms. Ng et al. [18] recruited 63 parents who had a preschool child diagnosed with autism spectrum disorder (ASD) in Hong Kong. They investigated the affiliate stigma among the parents and their children’s participations. Their findings indicated that parents having a preschool child with ASD had relatively high level of affiliate stigma. However, higher levels of affiliate stigma were not associated with most of the children’s participation. The negative association between parents’ affiliate stigma and children’s participation was only found in the community activities.

The affiliate stigma has also been studied in older populations. Su and Chang [19] assessed affiliate stigma and related psychological factors among family caregivers of people with dementia (n = 270) in Taiwan. Their findings indicate that affiliate stigma is associated with the psychological factors of anxiety and caregiving burden but not depression. In addition, male caregivers were found to have a higher level of affiliate stigma than their female counterparts. 

Lee et al. [20] assessed two types of stigma (public stigma and self-stigma) among 184 nurses who were at risk of depression in Korea. They found that public stigma, self-stigma, and depression are negatively associated with help-seeking behaviors. Moreover, self-stigma and depression were found to be significant mediators in the relationship between public stigma and seeking psychiatric help for depression problems. Therefore, they concluded the importance of regularly assessing self-stigma and depression levels among registered nurses.

Hanlon and Swords [21] invited 242 adolescents in Ireland to read a vignette describing a character (either a male (Sean) or a female (Katie)) with the problems of generalized anxiety disorder. Then, Hanlon and Swords [21] assessed the factors of stereotype, prejudice, discrimination, and help-giving intentions among these adolescents. They found that the endorsement of the stereotype of “weak-not-sick (i.e., the features of generalized anxiety disorder)” was associated with higher prejudice, stronger discrimination, and weaker intentions to help.

Ma and Hsieh [22] designed an anti-stigma course using a course framework (i.e., proceeding from knowledge to experience; then, action) to examine whether the anti-stigma course decreased the public stigma among occupational therapy students in Taiwan (n = 16). The outcome measures to assess the effectiveness of the anti-stigma course included two types of public stigma (discrimination through Social Distance Scale and attitudes through Stigmatizing Attitudes Toward Mental Illness). They found that the anti-stigma course had the effect of decreasing public stigma for the occupational students and such effects could last a year. 

Mahsoon et al. [23] used a cross-sectional design to understand how young adults in Saudi Arabia (n = 236) view the disease of mental illness and those with mental illness. Their results indicated that young adults in general had negative attitudes toward both the illness and people suffering from mental illness. In addition, beliefs toward mental illness, perceived parental support, and mental help-seeking attitudes were not associated in their study findings. Another Saudi Arabia study was a qualitative study on affiliate stigma. Specifically, Sharif et al. [24] utilized a qualitative study design with semi-structured interviews to assess affiliate stigma among family caregivers of people with mental illness (n = 13) in Saudi Arabia. They found that different types of burden experienced by the caregivers were highly needed after conducting the thematic analysis. Moreover, misconceptions about mental illness were found to be harmful for the caregivers and thus correcting such misconceptions is an important topic. 

Paananen et al. [25] used a qualitative study with a large sample size (n = 624 from mental health professionals and n = 300 dyads from mental health rehabilitants and their family members) to investigate the evaluation and rationale of stigma prevalence in mental health professionals and rehabilitants from the clubhouses in Finland. Through inductive content analysis, they found that perceived stigma is a phenomenon experienced by most of the rehabilitants. 

### 3.3. Stigma on Other Conditions (Homosexuality, Transgender, Physical Disability, Smoking, Solitude, Media Coverage of Pedophilia, and Public Housing Residents)

Ko et al. [26] used an online survey to assess the perceived stigma toward people who are homosexual and involved in same-sex marriage in two important time points in Taiwan: 23 months before the same-sex marriage referendum (Wave 1) and 2 months after the referendum (Wave 2). Among the non-heterosexual individuals (n = 798 in Wave 1 and 539 in Wave 2), their perceived unfavorable attitudes were associated with their suicidal ideation. Among heterosexual individuals (n = 1796 in Wave 1 and 1443 in Wave 2), those who were friendly with homosexual individuals (or supported the referendum) had the same association found in the non-heterosexual individuals (i.e., the perceived unfavorable attitudes toward homosexuality and same-sex marriage were associated with suicidal ideation). Similar to the context of homosexuality, Ozamiz-Etxebarria et al. [27] investigated the public stigma toward transgender people among university students in Spain. In general, the university students had relatively positive attitudes toward transgender people and this may be due to their higher education training. Nevertheless, the university students’ knowledge of transgender identity was not satisfactory. Therefore, Ozamiz-Etxebarria et al. [27] recommend the need to expand transgender education in the university environment. 

Silván-Ferrero et al. [28] invited 289 people with disabilities to participate in an online survey in Spain. The participants had different levels of disability, including those obtaining a Disability Certificate to access some benefits and those were qualified for non-contributory pension. Silván-Ferrero et al. [28] proposed a mediational path analysis and found that the association between higher levels of self-stigma and lower levels of quality of life among the participants with physical disability was mediated via resilience. Lewis et al. [29] utilized a qualitative design to understand the self-stigma of smoking among parents who had child(ren) at home in Scotland. Through thematic analysis, Lewis et al. [29] observed that parents may be afraid of disclosing their smoking habits in their societies because of their smoking self-stigma. Therefore, parents may perform hidden smoking and are not confident enough to stop smoking.

Lin et al. [30] treated solitude as a type of situation related to self-stigma and explored its relationship with physical and mental health among 562 junior college students in Taiwan. They found that the solitude capacity score was associated with physical and mental health. Therefore, they concluded that adolescents have better physical and mental health if they can cope with the self-stigma resulting from loneliness. Stelzmann et al. [31] used a qualitative study to investigate how healthcare providers in Germany view the media coverage of pedophilia. The healthcare providers included psychologists and medical doctors. In the semi-structured interview, both positive and negative attitudes toward the media coverage of pedophilia were found. The authors thus concluded that journalists may need to have a facto box to destigmatize pedophilia and increase the benefits of child sexual abuse prevention. Jun and Han [32] analyzed the data from the 2017 Seoul Public Housing Residents Panel Survey to investigate the experienced stigma issues among people who were living in public housing. They found that the association between discrimination and stress was lower in those living in social-mix housing complexes than in those living in independent public housing complexes. 

## 4. Conclusions and Future Directions

In conclusion, the importance of tackling stigma has been illustrated in a variety of populations. However, there is still a literature gap in the realm of stigma. Therefore, future studies are warranted to further explore and investigate this topic. Specifically, (1) the collected papers in the Special Issue used a cross-sectional design to investigate the phenomenon of stigma regardless of the type of stigma; (2) a number of papers in the Special Issue adopted a qualitative design to investigate the stigma phenomenon, which needs following quantitative evidence to support; (3) only one study examined the treatment effects on anti-stigmatization. Therefore, future studies could consider the following directions to explore in depth the issues of stigma in different populations: (1) using longitudinal designs to understand the temporal or causal relationship between stigma and other related psychosocial factors; (2) designing treatment programs to fight stigma—this could be carried out in healthcare providers, healthcare trainees, the public, caregivers, and stigmatized populations. 

## Figures and Tables

**Table 1:** The summarized findings among the collected 24 papers in the Special Issue.

Study/Country	Stigmatized Condition/Population	Participants’ Features	Methods	Type of Stigma	Measured Outcome	Findings
Lipowska et al. [9]/Poland and Vietnam	Body shape	University students (n = 1290; 586 from Poland (437 women; 149 men) and 704 (461 women; 246 men) from Vietnam; mean age = 20.36 to 21.77 years	Quantitative; cross-sectional	Perceived stigma	Body Esteem Scale; Perceived Stigmatization Questionnaire	University students in the collectivistic societies were vulnerable to criticisms of their bodies. Those from the Christian culture were not.
Lin et al. [10]/Taiwan	Obesity	Junior high school students (n = 464; 289 actual non-overweight and 176 actual overweight; 248 perceived non-overweight and 213 perceived overweight); mean age = 14.1 years	Quantitative; cross-sectional	Public stigma; perceived stigma; self-stigma	Perceived Weight Stigma Questionnaire; Weight Bias Internalization Scale; Beliefs About Obese Persons Scale; Hospital Anxiety Depression Scale	Adolescents with overweight regardless of actual or self-perceived had higher levels of perceived weight stigma and self-stigma than those without overweight. Perceived weight stigma was associated with anxiety among adolescents with overweight regardless of actual or self-perceived.
Fung et al. [11]/Hong Kong	Obesity	Young adults aged between 18 and 30 years (n = 325; 126 males and 199 females; 104 with overweight and 221 without overweight); mean age = 21.6 years.	Quantitative; cross-sectional	Self-stigma	International Physical Activity Questionnaire; Weight Bias Internalization Scale	Incorporated with the Theory of Planned Behavior, self-stigma was found to be negatively associated with physical activity level among young adults with overweight.
Wu et al. [12]/United States	Obesity	Asian Americans (n = 166; 92 males and 74 females); mean age = 45.7 years.	Quantitative; cross-sectional	Experienced stigma	Stigmatizing Situations Inventory; Binge Eating Scale; Suinn-Lew Asian Self-Identity Acculturation Scale; Subtle and Blatant Racism Scale; Perceived Stress Scale	Experienced weight stigma was found to be significantly associated with binge eating behaviors. Moreover, the aforementioned relationship controlled the confounding effects of racism and general stress.
Panza et al. [13]/United States	Obesity	Sexual minority women (n = 55; 62% bisexual or pansexual, 33% lesbian or homosexual, and 5% queer); mean age = 25 years.	Quantitative; cross-sectional	Experienced stigma	Stigmatizing Situations Inventory	The female sample with sexual minority had high rates of experienced weight stigma. In addition, they frequently reported co-occurrence of perceived and experienced weight stigma because of their marginalized identities.
Luís et al. [14]/Portugal	Postpartum mood and anxiety disorders	Male adults having a heterosexual relationship with a reproductive-age partner (n = 214); mean age = 32.61 years	Quantitative; cross-sectional	Not specified; possibly self-stigma	Gender-Role Conflict Scale; Acceptance and Action Questionnaire-II; Inventory of Attitudes Toward Seeking Mental Health Services; General Hel-Seeking Questionnaire	Male adults’ stigma may lower their intentions to recommend their partners with postpartum mood and anxiety disorders to seek professional help.
Rodríguez-Almagro et al. [15]/Spain	Mental illness	Spanish nursing students. Quantitative study: n = 359; 61 males and 298 females; mean age = 20 years. Qualitative study: n = 30; mean age = 20.2 years	Mixed-methods; cross-sectional	Public stigma	Mental Health Stigma Scale	Nursing students in a higher year of study had lower levels of public stigma toward mental illness than those in a lower year of study. The lowered public stigma was associated with more positive attitudes toward people with mental illness.
Riffel and Chen [16]/Canada	Mental illness	Healthcare students (n = 18 from nine healthcare programs; 12 females and 6 males)	Qualitative; qualitative description; semi-structure interview	Public stigma	--	Most students had good knowledge of mental health and they showed positive behaviors when interacting with people with mental illness. However, stigmatizing attitudes and unpreparedness to work with people with mental illness were observed in some students.
Chang et al. [17]/Taiwan	Attention-deficit/ hyperactivity disorder (ADHD)	Caregivers of children with ADHD (n = 400; 287 mothers, 90 fathers, and 23 others); mean age = 43.4 years.	Quantitative; cross-sectional	Affiliate stigma	Affiliate Stigma Scale; Caregivers’ Attitudes toward Children’s ADHD; Swanson, Nolan, and Pelham, version IV scale Parent Form	Higher levels of affiliate stigma were associated with caregivers’ unfavorable attitudes toward the diagnosis of ADHD and related treatments.
Ng et al. [18]/Hong Kong	Autism spectrum disorder (ASD)	Parents of preschool children with ASD (n = 63; 15 fathers, and 48 mothers); mean age = 39.1 years.	Quantitative; cross-sectional	Affiliate stigma	Young Children’s Participation and Environment Measure; Affiliate Stigma Scale	Parents of ASD children reported affiliate stigma above the midpoint of the Affiliate Stigma Scale. Parents’ affiliate stigma was not correlated with the children’s participation in frequency and involvement. However, parents’ affiliate stigma was associated with reduced involvement of their children in community activities.
Su and Chang [19]/Taiwan	Dementia	Family caregivers of people with dementia (n = 270; 142 females and 128 males; 167 were children of the care recipients and 36 were spouses of the care recipients); mean age = 52.3 years	Quantitative; cross-sectional	Affiliate stigma	Caregiver Burden Inventory; Affiliate Stigma Scale; Taiwanese Depressive Questionnaire; Beck Anxiety Inventory	Affiliate stigma of the family caregivers who took care of people with dementia was associated with higher levels of anxiety and caregiving burden. Moreover, male caregivers as compared with female caregivers had higher levels of affiliate stigma.
Lee et al. [20]/Korea	Depression	Registered nurses (n = 184; 10 males and 174 females); mean age = 26.6 years.	Quantitative; cross-sectional	Self-stigma; public stigma	Attitude Toward Psychiatric Help; Beck Depression Inventory-II; Depression Stigma Scale	The relationship between public stigma and attitudes toward seeking psychiatric help is mediated via self-stigma and depression.
Hanlon and Swords [21]/Ireland	Generalized anxiety disorder	Adolescents (n = 242; 74 males, 165 females, and 3 others); mean age = 16.5 years.	Quantitative; cross-sectional	Public stigma	Personal Depression Stigma Scale; Nine-item Emotional-ratings Scale; Social Distance Scale; Help-Giving Intentions	Endorsement of the stereotype of “weak-not-sick (i.e., the features of generalized anxiety disorder)” was associated with higher prejudice, stronger discrimination, and weaker intentions to help.
Ma and Hsieh [22]/Taiwan	Mental illness, emotional behavioral disorders, physical disability, and intellectual disability	Occupational therapy students (n = 16; 8 females and 8 males); mean age = 20.3 years at baseline	Quantitative; quasi-experimental (baseline, post-test, and one-year follow-up)	Public stigma	Social Distance Scale; Questionnaire on Stigmatizing Attitudes Toward Mental Illness	Occupational therapy students who completed an anti-stigma course had significantly decreased social distance and stigmatizing attitudes toward the stigmatized population. The decreased public stigma was maintained for a year.
Mahsoon et al. [23]/Saudi Arabia	Mental illness	Young adults (n = 236; 204 females and 32 males); age range = 18 to 25 years.	Quantitative; cross-sectional	Public stigma	Perceived Parental Support Scale; Mental Health Seeking Attitude Scale; Beliefs Toward Mental Illness Scale	The sample of young adults had relatively negative attitudes toward mental illness and people with mental illness. Moreover, beliefs toward mental illness, perceived parental support, and mental help-seeking attitudes were not associated.
Sharif et al. [24]/Saudi Arabia	Mental illness	Family caregivers of people with mental illness (n = 13; 77% females; age range = 21–65 years; 38% siblings, 31% parents, 15% children, and 8% spouses)	Qualitative; semi-structured interview	Affiliate stigma	--	Different types of burden experienced by the caregivers were highly needed. Misconceptions of mental illness were found to be harmful for the caregivers.
Paananen et al. [25]/Finland	Mental illness	Mental health professionals (n = 624) and mental health rehabilitants and their family member (n = 300 dyads) from the clubhouses.	Qualitative; video-recorded focus group interview	Perceived stigma	--	Perceived stigma is a phenomenon concerning most of the rehabilitants.
Ko et al. [26]/Taiwan	Homosexuality and same-sex marriage	Non-heterosexual individuals (n = 1796 (879 males and 917 females; mean age = 29.1 years) in Wave 1 and 798 (386 males and 412 females; mean age = 29.5 years) in Wave 2) and heterosexual individuals (n = 1443 (311 males and 1132 females; mean age = 32.4 years) in Wave 1 and 539 (123 males and 416 females; mean age = 36.3 years) in Wave 2)	Quantitative; two cross-sectional (Wave 1 at 23 months before same-sex marriage referendums in Taiwan; Wave 2 at one week after the referendums)	Perceived stigma	Suicidal Ideation; Unfavorable Attitudes toward Homosexuality and Same-Sex Marriage	Perceived unfavorable attitudes were associated with suicidal ideation among heterosexual individuals and heterosexual individuals who were friendly with homosexual individuals.
Ozamiz-Etxebarria et al. [27]/Spain	Transgender people	Undergraduate students (n = 376; 240 females, 117 males, and 19 others); mean age = 21.9 years.	Quantitative; cross-sectional	Public stigma	Gender and Transphobia Scale	University students had relatively positive attitudes toward transgender people; however, their knowledge of transgender identity needs to be improved.
Silván-Ferrero et al. [28]/Spain	Physical disability	People with physical disability (n = 289; 134 males and 155 females); mean age = 45.1 years.	Quantitative; cross-sectional	Self-stigma	Stigma Scale for Chronic Illness 9-item Version; Conner-Davidson Resilience Scale; WHO Quality of Life Questionnaire	The negative association between self-stigma and quality of life among people with physical disability was mediated via resilience.
Lewis et al. [29]/Scotland	Smoking	Parents who had child(ren) at home (n = 7; age range = 16 to 39 years; four single parents)	Qualitative; semi-structured interview	Self-stigma	--	Parents may be afraid of disclosing their smoking habits in their societies because of their smoking self-stigma. Therefore, parents may perform hidden smoking and are not confident to stop smoking.
Lin et al. [30]/Taiwan	Solitude	Junior college students (n = 562; 267 boys and 295 girls); mean age = 17.6 years	Quantitative; cross-sectional	Not specified; possibly self-stigma	Physical and Mental Health Scale; Solitude Capacity Scale	The junior college students reported that their solitude capacity was significantly associated with both physical and mental health.
Stelzmann et al. [31]/Germany	Media coverage of pedophilia	Healthcare providers (n = 11; seven psychologists and four medical doctors; five females and six males); mean age = 36.2 years	Qualitative; semi-structured interview	Public stigma	--	Positive attitude toward the media coverage of pedophilia has been stated in the qualitative study. However, public stigma of the media coverage of pedophilia has also been revealed among the healthcare providers.
Jun and Han [32]/Korea	Public housing residents	Public housing residents in Seoul (n = 4574; 1898 in social-mix housing complex and 2676 in independent public housing complex; 66% males); mean age = 61.8 years.	Quantitative; using the 2017 Seoul Public Housing Residents Panel Study	Not specified; possibly experienced stigma	One item on perceived stress (4-point Likert scale) and one item on discrimination (yes or no)	The association between discrimination and stress was lower in those living in social-mix housing complexes than in those living in independent public housing complexes.
